# Nitrate Increases Cadmium Accumulation in Sweet Sorghum for Improving Phytoextraction Efficiency Rather Than Ammonium

**DOI:** 10.3389/fpls.2021.643116

**Published:** 2021-05-20

**Authors:** Zhenqing Bai, Dan Li, Lin Zhu, Xiaoyu Tang, Yanfeng Wang, Renjun Mao, Jiawen Wu

**Affiliations:** ^1^Shaanxi Key Laboratory of Chinese Jujube, Yan’an University, Yan’an, China; ^2^College of Life Sciences, Yan’an University, Yan’an, China

**Keywords:** biomass, Cd, nitrogen, pH value, phytoremediation

## Abstract

Sweet sorghum has potential for phytoextraction of cadmium (Cd) owning to its large biomass and relatively high Cd tolerance. Nitrogen affects both growth and Cd concentrations in plants. However, different forms of nitrogen effects on Cd accumulation in sweet sorghum to improve efficiency of Cd phytoremediation is still elusive. In this study, nitrate substantially promoted both dry weight and Cd concentrations in leaves, stems + sheaths and roots of sweet sorghum when compared with ammonium. As a result, Cd accumulation in nitrate-supplied sweet sorghum was around 3.7-fold of that in ammonium-supplied plants under unbuffered pH condition, while the fold was about 2.2 under buffered pH condition. We speculated pH values and Cd species in the growth medium to some extent contributed to increased Cd accumulation as affected by nitrate. Net photosynthesis rate and Fv/Fm of nitrate-treated plants under Cd stress were higher than that of ammonium-treated plants when the pH was unbuffered. Responses of antioxidant capacity in roots to Cd stress with nitrate application were stronger than that with ammonium supplementation. Taken together, nitrate is more suitable than ammonium for Cd phytoextraction by using sweet sorghum, which is able to enhance at least double efficiency of phytoextraction.

## Introduction

Cadmium (Cd) contamination in soils including arable land is increasingly concerned worldwide as foundry, mining, smelting, sewage sludge application, indiscriminate supply of pesticide and low-grade fertilizers inevitably introduce Cd into pedosphere (Nagajyoti et al., 2010; Wu et al., 2019). Cd pollution in arable land generally shows a low to moderate degree of Cd pollution with a vast of polluted areas (Clemens et al., 2013; Shi et al., 2019). In moderately Cd-polluted soils, the Cd concentration in pore water is normally less than 0.5 μM, while this level is below 0.1 μM if amendments are added in soils (Quintela-Sabarís et al., 2017; Cornu et al., 2020). By contrast, numerous published papers studied Cd toxicity in plants by using 50–100 μM Cd concentrations which are hundreds times higher than that in pore water of Cd-contaminated soils. Thus, it is necessary to study response of plants to Cd stress that is lower than the level of 0.5 μM. Additionally, in the most of cases, low concentration of Cd in soils is negligible since this level of pollutant shows almost no harmful symptoms for crops growth and yields (Yan B.F. et al., 2019; Viala et al., 2021). However, if edible organs of crops harvested from Cd-polluted soils, even with a low amount of Cd, are consumed via food chain, human health will be damaged due to the enrichment of this heavy metal in bodies (Bernard, 2008). Therefore, strategies are required to decontaminate Cd in soils, while phytoremediation is the most suitable strategy to cope with Cd-polluted arable land.

Phytoextraction belongs to one of phytoremediation scenarios, which is a technique by using plants to remove Cd from soils and concentrate this pollutant into shoots of plants (Wu et al., 2018b; Mishra et al., 2019). Subsequently, the harvested shoots are buried, burned, composted or re-extracted for collecting heavy metal. Accordingly, plants used for Cd phytoextraction should deploy large biomass or tolerate high Cd concentration or both to improve the efficiency of remediation (de Sousa Leite and Monteiro, 2019). Although the well-known or newly discovered Cd hyperaccumulator plants such as *Sedum alfredii*, *Sedum plumbizincicola*, *Solanum nigrum* L., *Viola baoshanensis*, *Lantana camara* L., *Noccaea caerulescens*, etc. can endure high Cd concentrations without toxic symptoms, they normally have slow growth rate and small biomass (Li et al., 2018; Liu et al., 2019), thus limiting the efficiency of phytoremediation. By contrast, large biomass plants are able to accumulate high amounts of Cd although they are not Cd hyperaccumulators. In this regard, sorghum (*Sorghum bicolor* (L.) Moench.) is a C4 plant with high biomass and rapid growth rate, whilst sweet sorghum belongs to one variant of sorghum which has high sucrose content that is excellent feedstock for production of bioethanol (Mathur et al., 2017; Liu et al., 2020). By using sweet sorghum, a bioenergy plant, to remediate Cd-polluted agricultural soils, Cd will transfer from food chain to energy chain which could ensure efficacy of phytoextraction polluted-soils and economic incomes simultaneously (Grzegórska et al., 2020; Liu et al., 2020). Moreover, sweet sorghum performs better to tolerate Cd stress than maize (*Zea mays* L.) and wheat (*Triticum aestivum* L.) (Metwali et al., 2013). Zhuang et al. (2009) also found that sorghum accumulated more Cd in plants than sunflower (*Helianthus annuus* L.) another high biomass plant. Lyubun et al. (2020) indicated sorghum was a potential phytoremediation plant for not only Cd pollution but also oil sludge.

To further improve the efficiency of Cd phytoextraction, enlargement of plant biomass and/or enhancement of Cd concentrations in plants are beneficial. Nitrogen as the largest amount element after carbon required by plants has been well-known to be related to plant growth and Cd tolerance (Kovacik et al., 2011; Yang et al., 2020). However, the most two important forms of nitrogen taken up by roots, nitrate and ammonium distinctly regulate Cd accumulation in plants. For example, ammonium accelerates Cd accumulation in wheat (*T. aestivum* L.) (Yu et al., 2019), *Populus* clones (Bi et al., 2020), two Cd hyperaccumulators *Carpobrotus rossii* and *S. nigrum* (Cheng et al., 2016), whereas nitrate facilitates Cd accumulation in tomato (*Solanum lycopersicum*) (Luo et al., 2012), rice (*Oryza.sativa* L.) (Yang et al., 2016), Cd hyperaccumulators *N. caerulescens* and *S. plumbizincicola* (Hu et al., 2013). Wang et al. (2021) reported that absorption of ammonium with the root proton efflux decreased rhizosphere pH resulting in remobilized Cd in soils and increased Cd accumulation in amaranth (*Amaranthus mangostanus* L.). Zeng et al. (2020) indicated that ammonium increased Cd accumulation in *Brassica napus* as a result of decreased rhizosphere pH under a relatively low level of Cd treatment, whereas nitrate increased Cd accumulation in plants because of high biomass under a relatively high level of Cd treatment. Based on those findings, rhizosphere pH might play a crucial role in Cd accumulation in plants as affected by ammonium and nitrate. To elucidate effects of different nitrogen forms on plant growth and Cd concentrations in the large biomass plant sweet sorghum for enhancing the efficiency of phytoextraction, in the present study, buffered and unbuffered pH conditions were designed to investigate changes of Cd speciation in the growth medium, biomass, Cd accumulation, and physiochemical reactions such as antioxidant defense system and photosynthesis in ammonium or nitrate-suppled plants with or without Cd treatment.

## Materials and Methods

### Plant Cultivation and Treatments

Seeds of sweet sorghum cv. Dalishi were surface sterilized and then soaked in 0.5 mM CaCl_2_ with aeration for 8 h. Afterward, seeds with saturated water were germinated on three layers of moist filter papers. When the height of seedlings reached about 8 cm, uniform ones were transferred for hydroponics. To avoid osmotic stress, seedlings were cultivated in 1/4 strength nutrient solution for 3 days, and then 1/2 strength for 4 days. Subsequently, full-strength was used and Cd stress was started as well. Compositions of full-strength nutrient solution were macro-nutrient including K_2_SO_4_, 1.0 mM; KH_2_PO_4_, 0.2 mM; MgSO_4_, 0.5 mM and micro-nutrient including Fe-EDTA, 200 μM; H_3_BO_3_, 5 μM; MnSO_4_, 2 μM; ZnSO_4_, 0.5 μM; CuSO_4_, 0.3 μM; (NH_4_)_2_Mo_7_O_24_, 0.01 μM. Nitrogen source was supplied as 4 mM NO_3_^–^(N) in the form of Ca(NO_3_)_2_ or 4 mM NH_4_^+^ (A) in the form of NH_4_Cl, and nitrogen concentration was selected according to our previous study in sweet sorghum (Bai et al., 2021). To compensate the extra Ca^2+^ introduced by Ca(NO_3_)_2_, CaCl_2_ was added in the treatments without NO_3_^–^ addition. To be in accordance with the level of Cd concentration in pore water of most moderately Cd-polluted soils as stated in introduction section, 0.5 μM Cd in the form of CdCl_2_ was added in the present study, thus resulting in four treatments, N, N + Cd, A and A + Cd. Moreover, nutrient solution was divided into unbuffered and buffered pH two groups. Under buffered pH condition, at the beginning 10 days of Cd treatment, 2 mM 2-morpholinoethanesulfonic acid (MES) was added for keeping constant pH values among different treatments, and MES was increased to 5 mM between 11th and 20th day Cd addition. The concentration of MES was 10 mM after 20 days Cd treatment. In this study, Cd stress lasted 35 days and the changes of MES concentrations to buffer pH around 6.0 between nitrate and ammonium treatments were based on our preliminary experiment data. Four biological replicates were designed for each treatment, and one pot (two plants per pot) was regarded as one biological replicate. The whole experiment was conducted in the growth chamber with 28°C in the daytime, 20°C in the night, 75% humidity, a 14 h photoperiod and 350 μmol photon m^–2^ s^–1^ light intensity.

### Values of pH, Cd Speciation in the Growth Medium and Cd Accumulation Determination

Nutrient solution with unbuffered or buffered pH was changed every 5 days, and the renewed nutrient solution was always adjusted to 6.0 ± 0.1 with HCl and NaOH. The pH values in the unbuffered or buffered pH solution were determined at 10:00 in the morning every day until the end of 35 days Cd treatment.

Cd speciation in the growth medium with nitrate or ammonium supply was calculated by Visual MINTEQ 3.1 software. The concentrations of cations and anions individuals under 3.0, 4.0, 5.0, 6.0, and 7.0 pH were entered in the software for calculating proportions of different Cd species.

Cd accumulation was determined based on the result of dry weight multiplying Cd concentrations. In the present study, after 35 days Cd stress, sweet sorghum plants were divided into roots, stems + sheaths and leaves, dried at 75°C until constant weight, and then dry weight was measured. Afterward, the dried materials were milled into fine powder and then digested into ultra-pure HNO_3_ by a microwave oven (ETHOS UP, Milestone, Italy). Subsequently, the digested solution was diluted with 2% (v/v) HNO_3_ and then Cd concentrations were determined by inductively coupled plasma mass spectroscopy (ICP-MS, Agilent 7500, the United States) (Wu et al., 2016). It was worth mentioning that Cd concentrations in plants of non-Cd added treatments were not determined here since analytical grade chemicals and deionized water were used in the experiment. According to our preliminary study, Cd concentrations in those organs without Cd addition were below 0.05 μg g^–1^ dry weight which could be negligible when compared with high Cd concentrations in Cd-supplied plants (e.g., 30–120 μg g^–1^ dry weight in this experiment).

### Photosynthesis

The net photosynthesis rate (A), transpiration rate (E), stomatal conductance (Gs), intercellular CO_2_ concentration (Ci) and water use efficiency (WUE) of the third fully expanded leaves from bottom to top were determined by using a portable photosynthesis system (CIRAS-3, PP Systems, the United States). The parameters were measured between 9:00 and 11:00 in the morning.

Chlorophyll fluorescence parameters including the maximum efficiency of the PSII (Fv/Fm), photochemical quantum yield (QY), non-photochemical quenching (NPQ) and photochemical quenching (qP) of the third fully expanded leaves were measured by using a closed chlorophyll fluorescence imaging system (800MF, FluorCam, Czech Republic). Before measurement, the leaves were adapted in the darkness for at least 20 min, and then the plants avoiding light were transferred into the chamber of chlorophyll fluorescence imaging instrument for determining the chlorophyll fluorescence parameters (Singh et al., 2021).

### H_2_O_2_ Content Determination and Antioxidant Defense System

After Cd treatment, roots and the third fully expanded leaves were washed with deionized water at least three times, gently dried by tissue paper, quickly frozen in liquid N_2_ and then stored at −80°C for determining H_2_O_2_ contents, total antioxidants, superoxide dismutase (SOD), catalase (CAT), guaiacol peroxidase (POD) activities and reduced glutathione contents (GSH). The H_2_O_2_ contents and total antioxidants were determined according to our previous work (Wu et al., 2018a), in which the materials were homogenized in 0.1% (w/v) trichloroacetic acid (TCA) and then centrifuged at 12,000 g for 10 min at 4°C. The supernatant was incubated with 10 mM potassium phosphate buffer (pH 7.0) and 1 M potassium iodide (KI) for 15 min. Subsequently, OD values of incubated solution were determined at 390 nm by a spectrophotometer (F-4500, Hitachi, Japan), while H_2_O_2_ contents were calculated based on the standard curve of H_2_O_2_ contents. For measuring the total antioxidants, materials were homogenized in 50% (v/v) ethanol and then centrifuged at 12,000 g for 10 min at 4°C. Afterward, the supernatant was added with 99% (v/v) ethanol and 3 mM 1, 1-diphenyl-2-picrylhydrazyl radical (DPPH.) which should be freshly prepared in the darkness for 10 min. OD values of the reaction solution were determined at 515 nm. Positive control was the reaction with 99% (v/v) ethanol instead of supernatant from materials. The percentages of different absorbance between positive control and samples accounted for positive control were regarded as total antioxidants (Wu et al., 2018a).

Activities of SOD and CAT, and GSH contents were also determined according to our previous published paper (Wu et al., 2015). Briefly, SOD activity was determined based on nitroblue tetrazolium (NBT) method. CAT activity was determined based on enzyme kinetics of decomposing H_2_O_2_, while the absorbance was measured at 240 nm. GSH was determined based on dithiobis-2-nitrobenzoic acid (DTNB) method, in which cautions should take for finishing the determination at 412 nm in 5 min after the incubation. POD activity was determined by reagent kit (Solarbio) using guaiacol method, which was calculated based on enzyme kinetics of decomposing H_2_O_2_ at 470 nm.

### Statistical Analysis

All the data presented in this study was four replicates ± standard deviation (SD). Significant differences between N, N + Cd, A and A + Cd treatments were analyzed according to One-Way ANOVA at the level of 0.05, while the differences between N + Cd and A + Cd treatments were analyzed according to student *t*-test by using SPSS (16.0) software.

## Results

### Effects of Nitrogen Forms on Cd Species and pH Changes in the Growth Medium

**FIGURE 1:**
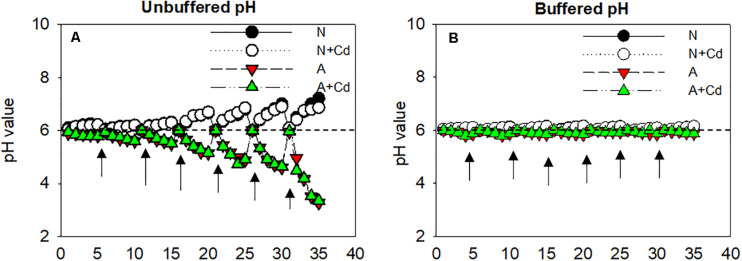
Changes of pH values in the growth medium as affected by nitrate and ammonium in the presence or absence of Cd under unbuffered **(A)** or buffered **(B)** pH condition. N, nitrate; N + Cd, nitrate with Cd; A, ammonium; A + Cd, ammonium with Cd. Arrows represented time points of renewing nutrient solution which was changed every 5 days.

As can been seen in Figure 1A, regardless of Cd addition, nitrate (N) remarkably alkalized the growth medium with the gradually increased pH values, whereas ammonium (A) acidified the growth medium with the gradually decreased pH values. Although nutrient solution was renewed every 5 days and adjusted the pH to 6 ± 0.1, the pH values in the growth medium were changed as the uptake of NH_4_^+^ or NO_3_^–^ by roots and the acidification or alkalization was sharper at the later growth stage. To distinguish whether pH in the root rhizosphere as affected by nitrate and ammonium played a pivotal role in Cd accumulation in sweet sorghum, MES was added in the nutrient solution to buffer constant pH values compared with unbuffered nutrient solution. In the buffered pH growth medium, pH values were 6 ± 0.1 during the whole cultivation period irrespective of nitrogen forms (Figure 1B).

In the present study, a chemical equilibrium model, Visual MINTEQ was used to estimate the proportions of different Cd speciation in the nutrient solution as affected by different nitrogen forms and pH values. According to Figure 1, changes of pH varied from about 3.0–7.0 influenced by nitrate and ammonium. Thus, Cd speciation in the growth medium was calculated from 3.0 to 7.0 under nitrate or ammonium with Cd treatment. When pH value in the nutrient solution was 7.0, Cd^2+^ and CdHPO_4_^0^ accounted for 76.96 and 12.56% under nitrate treatment, while the major species were Cd^2+^ and CdCl^+^ with 50.74 and 34.78%, respectively, under ammonium treatment (Table 1). Nevertheless, under 6.0, 5.0, 4.0, and 3.0 pH values, the largest two percentages of Cd speciation were Cd^2+^ with 85.68, 87.86, 88.16, and 89.02%, and CdSO_4_^0^ with 10.70, 10.96, 10.89, and 10.06% under nitrate supplementation. In contrast, in ammonium-treated growth medium, under 6.0, 5.0, 4.0, and 3.0 pH values, Cd^2+^ was the most important speciation with 54.12, 54.91, 55.02, and 55.41, while the second important speciation CdCl^+^ occupied 37.12, 37.66, 37.71, and 37.75%. Except aforementioned Cd speciation, CdOH^+^, CdCl_2_^0^, Cd(SO_4_)_2_^2–^, CdNO_3_^+^, and CdNH_3_^2+^ also existed in the growth medium, but they only contributed to minor proportions (Table 1).

**TABLE 1:** Distribution of Cd speciation as affected by nitrate and ammonium under different pH values in the growth medium.

pH	Treatments	Name of Cd speciation (% of total concentration)								
		Cd^2+^	CdOH^+^	CdCl^+^	CdCl_2_^0^	CdSO_4_^0^	Cd(SO_4_)_2_^2–^	CdNO_3_^+^	CdNH_3_^2+^	CdHPO_4_^0^
7.0	N + Cd	76.960	0.045	0.014	None	9.621	0.16	0.638	None	12.560
	A + Cd	50.748	0.028	34.788	1.338	5.317	0.089	None	0.360	7.330
6.0	N + Cd	85.688	None	0.016	None	10.703	0.178	0.711	None	2.698
	A + Cd	54.127	None	37.121	1.428	5.667	0.095	None	0.039	1.520
5.0	N + Cd	87.867	None	0.017	None	10.966	0.182	0.729	None	0.238
	A + Cd	54.910	None	37.662	1.449	5.745	0.096	None	None	0.133
4.0	N + Cd	88.162	None	0.017	None	10.896	0.180	0.730	None	0.014
	A + Cd	55.027	None	37.713	1.45	5.707	0.095	None	None	None
3.0	N + Cd	89.026	None	0.017	None	10.068	0.154	0.732	None	None
	A + Cd	55.414	None	37.753	1.447	5.302	0.082	None	None	None

*The “None” represented this type of Cd speciation was not found. Proportions of different Cd speciation in this table were calculated based on a chemical equilibrium software, Visual MINTEQ 3.1.*

### Nitrogen Forms Affect Biomass and Cd Accumulation in Sweet Sorghum Under Unbuffered and Buffered pH Conditions

**FIGURE 2:**
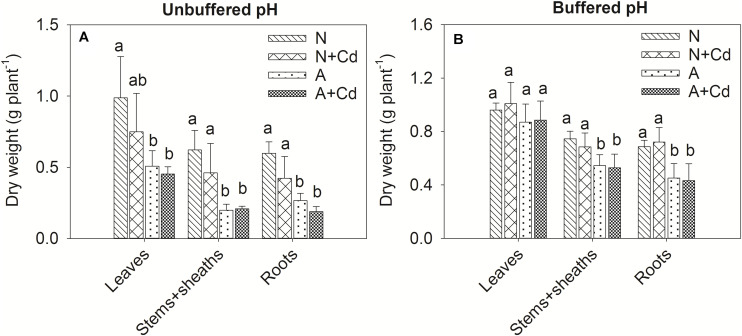
Dry weight of leaves, stems + sheaths and roots in sweet sorghum as affected by nitrate and ammonium in the presence or absence of Cd under unbuffered **(A)** or buffered **(B)** pH condition. N, nitrate; N + Cd, nitrate with Cd; A, ammonium; A + Cd, ammonium with Cd. Different letters above the bars of one organ represented significant differences at the level of 0.05 based on One-Way ANOVA and Duncan’s multiply analysis.

**FIGURE 3:**
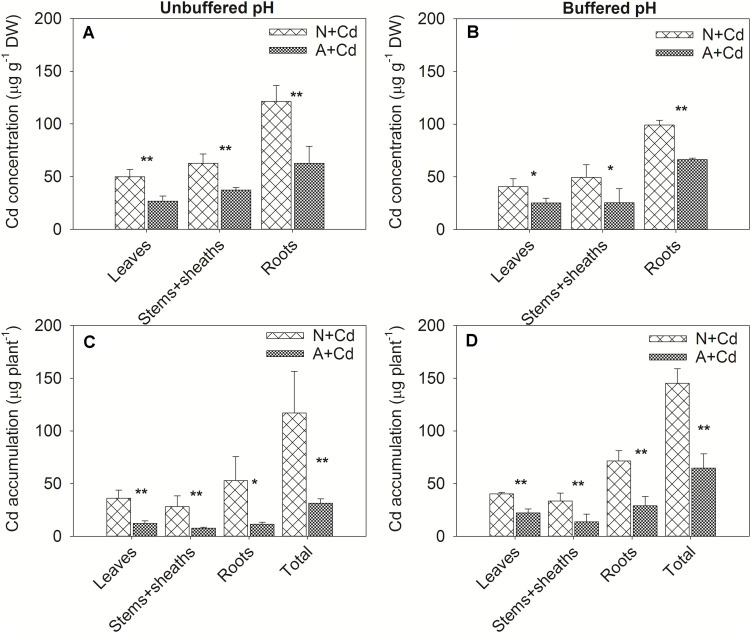
Cd concentrations and accumulation in leaves, stems + sheaths and roots of sweet sorghum as affected by nitrate and ammonium in the presence or absence of Cd under unbuffered **(A,C)** or buffered **(B,D)** pH condition. N + Cd, nitrate with Cd; A + Cd, ammonium with Cd. One asterisk “*” indicated significant difference between the two treatments at the level of 0.05 based on student *t*-test, while two asterisks “**” represented at the level of 0.01.

In this study, a low concentration of 0.5 μM Cd was conducted since Cd contamination in agricultural soils usually shows thus low concentration of pollution. Dry weight of leaves, stems + sheaths and roots in sweet sorghum was not significantly affected by Cd stress regardless of nitrogen forms or pH in the growth medium (Figure 2). However, under unbuffered pH condition, dry weight of leaves, stems + sheaths and roots in nitrate-treated plants was substantially higher than that in ammonium-treated plants (Figure 2A). Under buffered pH condition, dry weight of stems + sheaths and roots with nitrate supply was also higher than that with ammonium supply, whereas dry weight of leaves was similar between two different nitrogen forms treatments (Figure 2B). With regard to Cd concentrations, under both unbuffered and buffered pH conditions, Cd concentrations in leaves, stems + sheaths and roots with nitrate treatment were drastically increased when compared with ammonium-treated plants (Figures 3A,B). Based on the data of dry weight and Cd concentrations, under unbuffered pH condition, Cd accumulation amounts in leaves, stems + sheaths, roots and total plant per sweet sorghum with nitrate supply were about 2. 98-, 3. 62-, 4. 60-, and 3.73-fold of that in ammonium-treated plants (Figure 3C). Under buffered pH condition, Cd accumulation values in nitrate-treated plants were 1. 82-, 2. 46-, 2. 47-, and 2.25-fold in leaves, stems + sheaths, roots and total plant of that in ammonium-treated plants, respectively (Figure 3D).

### Nitrogen Forms Affect Photosynthesis and Antioxidant Defense System in Sweet Sorghum Under Unbuffered and Buffered pH Conditions

**FIGURE 4:**
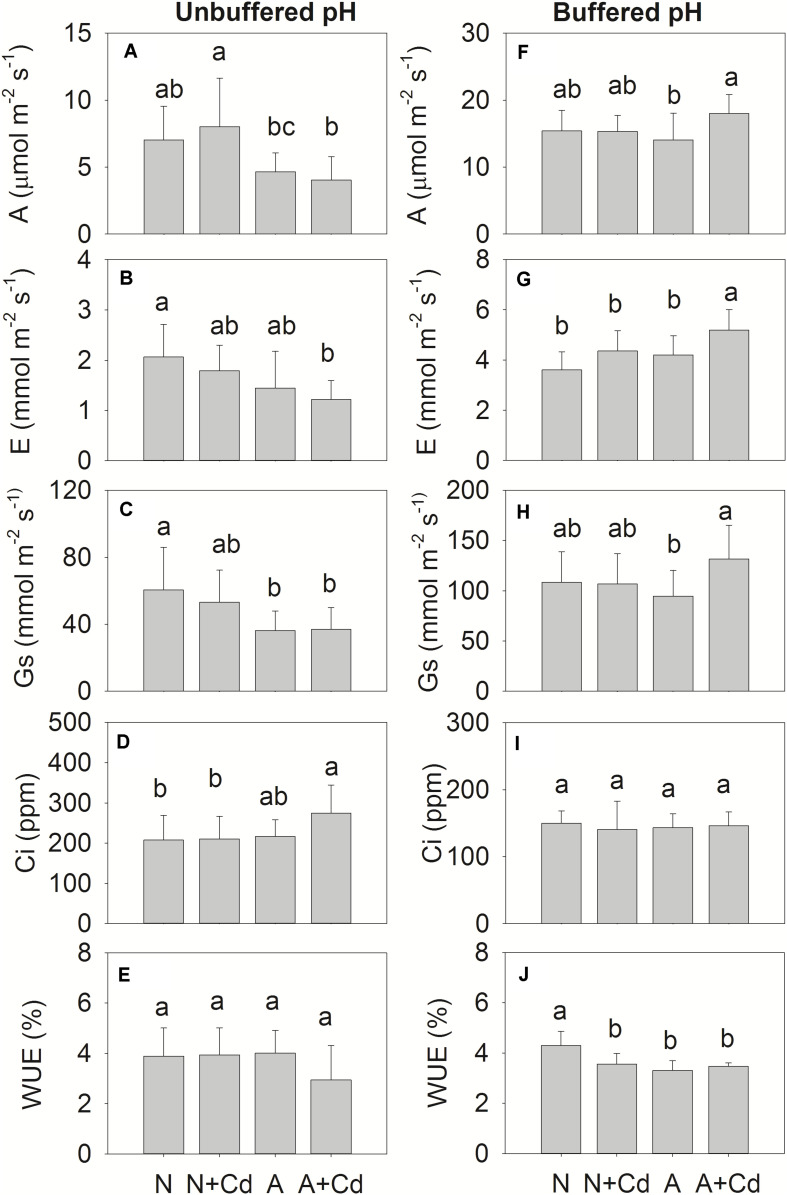
Net photosynthesis rate (A), transpiration rate (E), stomatal conductance (Gs), intercellular CO_2_ concentration (Ci) and water use efficiency (WUE) in leaves of sweet sorghum as affected by nitrate and ammonium in the presence or absence of Cd under unbuffered **(A–E)** or buffered **(F–J)** pH condition. N, nitrate; N + Cd, nitrate with Cd; A, ammonium; A + Cd, ammonium with Cd. Different letters above the bars represented significant differences at the level of 0.05 based on One-Way ANOVA and Duncan’s multiply analysis.

**FIGURE 5:**
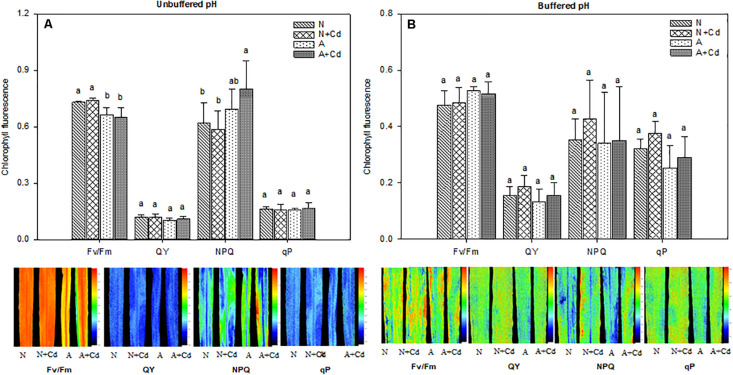
Maximum efficiency of the PSII (Fv/Fm), photochemical quantum yield (QY), non-photochemical quenching (NPQ) and photochemical quenching (qP) in leaves of sweet sorghum as affected by nitrate and ammonium in the presence or absence of Cd under unbuffered **(A)** or buffered **(B)** pH condition. N, nitrate; N + Cd, nitrate with Cd; A, ammonium; A + Cd, ammonium with Cd. Different letters above the bars represented significant differences at the level of 0.05 based on One-Way ANOVA and Duncan’s multiply analysis.

Net photosynthesis rate (A), transpiration rate (E), stomatal conductance (Gs), intercellular CO_2_ concentration (Ci), water use efficiency (WUE), and parameters regarding fluorescence of photosynthesis such as Fv/Fm, QY, NPQ and qP were determined in this study. Under unbuffered pH condition, in accordance with the results of dry weight (Figure 2A), Cd stress had no effects on A, E, Gs, Ci and WUE when compared with their corresponding nitrogen treatments (Figures 4A–E), which indicated the same trends for Fv/Fm, QY, NPQ and qP (Figure 5A). Compared with ammonium supply under Cd addition, nitrate significantly increased A and decreased Ci in Cd-stressed sweet sorghum plants (Figures 4A,D). Under buffered pH condition, in nitrate-added plants, Cd stress had no significant effects on A, E, Gs and Ci except declined WUE compared with non-Cd treatment (Figures 4F–J), while Fv/Fm, QY, NPQ, and qP were not affected either (Figure 5B). Nevertheless, in ammonium-added plants, Cd stress remarkably elevated A, E and Gs with unchanged Ci and WUE when compared with non-Cd treatment (Figures 4F–J), whereas their fluorescence parameters of photosynthesis Fv/Fm, QY, NPQ, and qP were not changed (Figure 5B).

In regard to oxidative stress and antioxidant defense system, under unbuffered pH condition, ammonium significantly increased H_2_O_2_ contents in leaves of sweet sorghum compared with nitrate when no Cd was added, while total antioxidants, SOD, CAT, POD activities, and GSH contents were not changed between two nitrogen forms. Under Cd stress, the low level of Cd in the present study had no effects on H_2_O_2_ production and anti-oxidases activities in leaves with nitrate application, whereas Cd decreased CAT activity with ammonium application when other antioxidant enzymes were unaffected compared with non-Cd added treatment (Table 2). Interestingly, Cd stress drastically decreased H_2_O_2_ contents and SOD activity, but increased GSH contents in nitrate-treated roots of sweet sorghum. In contrast, Cd stress also elevated GSH contents, while H_2_O_2_ contents, total antioxidants, SOD, CAT and POD activities were not affected in ammonium-treated roots of plants when compared with non-Cd addition (Table 2). Under buffered pH condition, Cd stress significantly increased CAT and POD activities in nitrate-supplied leaves of plants compared with non-Cd added treatment, while Cd exposure had no effects on all the determined antioxidant enzymes activities with ammonium supply (Table 3). In roots, nitrate application significantly decreased total antioxidants, SOD and POD activities under Cd stress when compared with non-Cd added treatment, whereas ammonium increased SOD activity under Cd stress with no changes on other antioxidant enzymes activities (Table 3).

**TABLE 2:** Effects of nitrate and ammonium on antioxidant defense systems in leaves and roots of sweet sorghum with or with Cd stress under unbuffered pH condition.

Organ	Treatment	H_2_O_2_ content (μ mol g^–^^1^ FW)	Total antioxidants (%)	SOD activityU g^–^^1^ FW)	CAT activity (Δ 240 μ g protein^–^^1^ min^–^^1^)	POD activity (Δ 470 μ g protein^–^^1^ min^–^^1^)	GSH content (μ g g^–^^1^ FW)
Leaves	N	128.1 ± 40.8^b^	28.0 ± 5.9^a^	197.4 ± 27.4^a^	480.1 ± 246.8^ab^	89.3 ± 19.2^a^	92.2 ± 5.2^a^
	N + Cd	141.7 ± 30.1^b^	36.3 ± 6.3^a^	192.9 ± 50.0^a^	228.5 ± 130.2^b^	71.9 ± 8.2^ab^	133.2 ± 47.7^a^
	A	248.5 ± 58.4^a^	35.4 ± 7.8^a^	208.4 ± 25.7^a^	696.0 ± 131.0^a^	68.8 ± 15.3^ab^	126.5 ± 29.9^a^
	A + Cd	183.2 ± 43.6^ab^	33.2 ± 5.9^a^	197.1 ± 34.5^a^	289.1 ± 153.8^b^	54.1 ± 12.1^b^	125.4 ± 14.3^a^
Roots	N	70.7 ± 30.7^a^	16.7 ± 2.6^ab^	218.1 ± 15.9^a^	139.1 ± 30.7^a^	538.4 ± 174.5^a^	25.6 ± 8.9^b^
	N + Cd	40.1 ± 9.0^b^	21.9 ± 5.7^a^	145.2 ± 11.4^b^	106.5 ± 35.2^ab^	533.7 ± 59.0^a^	99.4 ± 10.8^a^
	A	37.3 ± 11.6^b^	15.7 ± 2.4^b^	151.1 ± 45.3^b^	77.9 ± 43.0^b^	391.9 ± 80.6^a^	36.6 ± 13.2^b^
	A + Cd	38.3 ± 4.8^b^	17.8 ± 1.8^ab^	146.2 ± 28.5^b^	81.8 ± 10.8^b^	398.9 ± 82.6^a^	87.3 ± 17.8^a^

*Values were means ± SD (n = 4). Different letters in the same column of leaves or roots represented significant differences at the level of 0.05 based on One-Way ANOVA and Duncan’s multiply analysis. N, nitrate; N + Cd, nitrate with Cd; A, ammonium; A + Cd, ammonium with Cd.*

**TABLE 3:** Effects of nitrate and ammonium on antioxidant defense systems in leaves and roots of sweet sorghum with or without Cd stress under buffered pH condition.

Organ	Treatment	H_2_O_2_ content (μ mol g^–^^1^ FW)	Total antioxidants (%)	SOD activity (U g^–^^1^ FW)	CAT activity (Δ 240 μ g protein^–^^1^ min^–^^1^)	POD activity (Δ 470 μ g protein^–^^1^ min^–^^1^)	GSH content (μ g g^–^^1^ FW)
Leaves	N	173.8 ± 83.9^a^	20.9 ± 9.1^a^	145.3 ± 41.5^a^	84.9 ± 24.4^b^	103.1 ± 13.9^b^	82.6 ± 39.5^a^
	N + Cd	122.1 ± 39.1^a^	11.6 ± 4.5^a^	183.6 ± 18.3^a^	176.3 ± 74.5^a^	176.4 ± 65.9^a^	86.7 ± 14.8^a^
	A	181.1 ± 43.1^a^	19.9 ± 3.9^a^	157.4 ± 15.7^a^	100.2 ± 31.7^ab^	73.5 ± 20.7^b^	85.9 ± 20.6^a^
	A + Cd	223.2 ± 77.9^a^	19.0 ± 6.6^a^	178.1 ± 13.3^a^	134.4 ± 59.5^ab^	112.7 ± 37.7^b^	110.7 ± 7.22^a^
Roots	N	55.9 ± 28.5^a^	6.25 ± 2.09^a^	131.2 ± 21.0^a^	29.9 ± 9.1^a^	500.9 ± 140.9^a^	18.9 ± 17.7^b^
	N + Cd	35.8 ± 10.7^ab^	3.51 ± 1.18^b^	23.2 ± 6.17^c^	42.9 ± 8.6^a^	203.1 ± 28.3^b^	41.2 ± 22.9^ab^
	A	28.4 ± 12.2^ab^	4.17 ± 1.64^ab^	37.4 ± 16.2^c^	39.3 ± 12.0^a^	437.1 ± 75.8^a^	46.6 ± 11.9^ab^
	A + Cd	27.6 ± 1.34^b^	3.42 ± 0.23^b^	63.1 ± 13.4^b^	46.9 ± 14.9^a^	409.4 ± 52.7^a^	54.3 ± 26.7^a^

*Values were means ± SD (n = 4). Different letters in the same column of leaves or roots represented significant differences at the level of 0.05 based on One-Way ANOVA and Duncan’s multiply analysis. N, nitrate; N + Cd, nitrate with Cd; A, ammonium; A + Cd, ammonium with Cd.*

## Discussion

Phytoextraction of Cd-polluted soils by high biomass crops such as sweet sorghum is increasingly concerned (Liu et al., 2020; Lyubun et al., 2020). Nitrogen management is not only one of important agronomic measures, but also a pivotal element determining biomass of plants (Hawkesford et al., 2012). Owing to effects of different nitrogen forms on Cd uptake, translocation and accumulation in different species of plants differently, how the two frequently used nitrogen forms, nitrate and ammonium functions on Cd accumulation in sweet sorghum are still unclear. Since nitrate and ammonium are physiologically alkaline and acidic fertilizers due to influx and efflux of protons in the growth medium separately (de Sousa Leite and Monteiro, 2019; Zeng et al., 2020), unbuffered and buffered conditions were conducted in the present study to exam pH effects on Cd accumulation in sweet sorghum treated with different forms of nitrogen. Empirically, when roots absorb one molecular NH_4_^+^, one molecular H^+^ will be pumped from cytosol into rhizosphere leading to a decrease of rhizosphere pH (Hawkesford et al., 2012), thus increasing bioavailability of Cd and accumulation of this toxic element in plants. However, our results showed that nitrate-supplied plants accumulated much higher Cd than ammonium-supplied plants regardless of pH conditions (Figure 3), representing pH was not the main factor influencing Cd accumulation as affected by nitrate and ammonium. This conclusion was consistent with the finding in *N. caerulescens* that nitrate compared with ammonium facilitated Cd and Zn accumulation irrespectively of rhizosphere pH (Xie et al., 2009). Considering speciation forms also affect Cd uptake by roots, different Cd species between pH 3.0 and pH 7.0 based on unbuffered pH conditions as affected by nitrate and ammonium were determined (Figure 1). When pH values decreased from 7.0 to 3.0, percentages of Cd species including Cd^2+^ were only slightly elevated with the exception of a decreased CdHPO_4_^0^ in nitrate-added growth medium. Cd species in ammonium-supplied growth medium were also slightly affected by different pH values. However, Cd^2+^ and CdSO_4_^0^ occupied the majority of Cd species ranging 76.9–89.0 and 9.6–10.1% in nitrate-treated growth medium, while Cd^2+^ and CdCl^+^ accounted for 50.7–55.4 and 34.8–37.7% in ammonium-treated growth medium (Table 1). Our previous studies found that CdSO_4_^0^ was easier to be taken up by roots than Cd^2+^ because of faster diffusion (Wu et al., 2018b). Weggler et al. (2004) showed that excessive supply of Cl^–^ significantly increased mobilization and bioavailability of Cd in soils, whereas Geilfus (2019) indicated Cl immobilized Cd availability because of Cd and Cl^–^ complexes formation. In view of chemical theory, diffusion of CdCl^+^ in roots should be faster than Cd^2+^ as binding ability of monovalent ions are weaker than divalent ions with functional groups such as carboxyl or hydroxyl in cell walls. Nevertheless, evaluating Cd bioavailability only by proportions of Cd species in the growth medium is still difficult to judge how much Cd accumulation in plants. On account of transporters like IRT1, Nramp1, Nramp5, HMA2, HMA3, and HMA4 etc. correlated with Cd^2+^ uptake and transport are well-known, but transporters for taking up CdCl^+^, CdSO_4_^0^ or other Cd-complex are unidentified in plants to date (Clemens et al., 2013). Cheng et al. (2016) reported neither ammonium nor nitrate had effects on existence of Cd speciation in *planta* of *C. rossii* and *S. nigrum*. Therefore, although Cd species in the growth medium are differently changed by nitrate and ammonium, their effects on Cd uptake and accumulation in plants are still limited.

Biomass and Cd concentration are two determinants of total Cd accumulation in plants that directly correlates with Cd phytoextraction efficiency. Vazquez et al. (2020) found ammonium supply increased plant biomass contributed to declined Cd concentrations in *Arabidopsis thaliana* because of “dilution effect,” which was also reported in wheat (Yu et al., 2019). Yang et al. (2019) showed (NH_4_)_2_SO_4_ and CH_4_N_2_O had no effect on Cd concentrations but increased plant biomass resulting in raised Cd accumulation in *S. nigrum*. Cheng et al. (2016) reported ammonium compared with nitrate increased Cd accumulation as the consequence of elevated Cd concentrations in *C. rossii* and *S. nigrum* although biomass was unchanged. Our results showed that nitrate compared with ammonium promoted both dry weight and Cd concentrations in sweet sorghum regardless of pH in the growth medium (Figures 2, 3). Accordingly, total Cd accumulation in nitrate-supplied plants were about 3.7- and 2.2-fold of that in ammonium-supplied plants under unbuffered and buffered pH, respectively, suggesting that nitrate enhances Cd phytoextraction efficiency when compared with ammonium.

Photosynthesis provides substrates for plant growth which is closely related to biomass (Alves et al., 2020). As a toxic element, Cd is able to damage photosynthesis apparatus leading to retarded plant growth (Nagajyoti et al., 2010). Based on majority of Cd concentration in pore water of Cd-contaminated soils (Quintela-Sabarís et al., 2017), 0.5 μM Cd was used in this study. Nonetheless, net photosynthesis rate and Fv/Fm were not decreased by Cd stress (Figures 4, 5) which contributed to unchanged dry weight of shoots and roots of sweet sorghum as affected by Cd addition in this study (Figure 2). In contrast, Cornu et al. (2020) showed that a lower level of 0.1 μM Cd exposure significantly decreased dry weight of sunflower. It seems that sweet sorghum is more Cd tolerant than sunflower although both of them are high biomass plants, indicating better potential of sweet sorghum for Cd phytoextraction. Yan L. et al. (2019) found ammonium tended to enhance roots rather than shoots growth of terrestrial plants, while nitrate preferred to promote shoots growth by using a meta-analysis, which suggests nitrate-treated plants have higher biomass of shoots that is more suitable for Cd phytoremediation. In the present study, nitrate increased dry weight of both roots and shoots of sweet sorghum (Figure 2), which was the result of nitrate-enhanced net photosynthesis rate and Fv/Fm under Cd stress compared with ammonium (Figures 4, 5). It was worth mentioning that Cd hormesis in the presence of ammonium deployed the ability of improving photosynthesis by increasing stomatal open and transpiration (Figure 4), and this kind of heavy metal hormesis was also reported in maize by Małkowski et al. (2020). Although Cd stimulated net photosynthesis rate under ammonium, the Fv/Fm was unchanged under buffered pH growth medium (Figures 4, 5), which might be the reason that dry weight of leaves between nitrate and ammonium treatments were same (Figure 2). Overall, photosynthesis rates of nitrate-supplied sweet sorghum are higher than that of ammonium-supplied plants especially under unbuffered pH condition. Regarding Cd toxicity, oxidative stress is one of the toxic biomarkers although this element dose not directly involve in Fenton or Haber-Weiss reactions producing ROS (reactive oxygen species) (Nagajyoti et al., 2010). Correspondingly, plants evolve enzymatic antioxidants such as SOD, CAT, POD, GR (glutathione reductase) or APX (ascorbate peroxidase) and non-enzymatic antioxidants such as AsA (ascorbate), GSH or phenolics to scavenge the excessive ROS that maintains redox homeostasis (Wu et al., 2015). In this study, compared with non-Cd added treatments H_2_O_2_ contents were not increased by nitrate or ammonium with Cd added treatment (Tables 2, 3), which indicated excessive ROS was not induced by Cd addition and further proved Cd tolerance of sweet sorghum. However, Lin et al. (2011) showed that high level of nitrogen compared with the low level significantly increased some antioxidant enzymes contributing to mitigation of Cd toxicity in rice. Jalloh et al. (2009) found rice plants supplied with ammonium had less oxidative stress than nitrate-supplied plants as induced by Cd stress, which was also confirmed by Wu et al. (2020) that ammonium enhanced antioxidant system and AsA-GSH cycle in rice. In contrast, Nogueirol et al. (2018) reported nitrate alleviated Cd-caused oxidative stress by improving CAT, APX, SOD, POD and GR activities in tomato. Bi et al. (2020) indicated nitrate significantly elevated POD, SOD and GR activities in leaves of Nanlin1388 *Populus* clone under Cd stress, while nitrate had no effects on those anti-oxidase enzymes activities in Nanlin 895. Thus, enzymatic and non-enzymatic antioxidants were differently regulated by nitrogen forms in different plant species under Cd stress. Our results showed that nitrate compared with ammonium significantly increased POD activity in leaves, but decreased SOD and POD activities in roots of sweet sorghum in the presence of Cd addition (Table 3). SOD could convert superoxide radicals (O_2_^–^) into H_2_O_2_, while POD is responsible for catalyzing H_2_O_2_ into water and oxygen (Wu et al., 2015). Increased POD activity in leaves as affected by nitrate under Cd stress in sweet sorghum indicated enhanced antioxidant capacity, and decreased SOD and POD activities in roots might be a consequence of low oxidative stress that antioxidant enzymes were not required for scavenging excessive ROS. Therefore, we speculate that anti-oxidative responses of roots in nitrate-supplied sweet sorghum to Cd addition are stronger than that of ammonium-supplied plants.

## Conclusion

Nitrate significantly increases Cd accumulation in roots, stems + sheaths and leaves of sweet sorghum when compared with ammonium as the consequence of enhanced both dry weight and Cd concentrations. Total Cd amounts in nitrate-treated sweet sorghum is about 3.7-fold of that in ammonium-treated plants under unbuffered pH condition, while it is 2.2-fold under buffered pH condition. We reason the pH and different proportions of Cd species in the growth medium to some extent contribute to improvement of Cd accumulation as affected by nitrate. In addition, nitrate elevates photosynthesis rate under Cd stress compared with ammonium especially under unbuffered pH condition, which might be the reason of promoted dry weight by nitrate. Anti-oxidative responses to Cd stress in nitrate-supplied roots are stronger than that in ammonium-supplied plants. Taken together, nitrate enhances Cd accumulation in sweet sorghum aiming at improvement of phytoextraction efficiency when compared with ammonium.

## Data Availability Statement

The original contributions presented in the study are included in the article/supplementary material, further inquiries can be directed to the corresponding author/s.

## Author Contributions

ZB, DL, LZ, and XT carried out the experiments. ZB and LZ wrote the manuscript with support from JW. DL analyzed data for this work and revised the manuscript with support from JW. YW and RM helped to supervise the project. JW conceived the original idea and supervised the project.

## Conflict of Interest

The authors declare that the research was conducted in the absence of any commercial or financial relationships that could be construed as a potential conflict of interest.
